# Taking multi-morbidity into account when attributing DALYs to risk factors: comparing dynamic modeling with the GBD2010 calculation method

**DOI:** 10.1186/s12889-017-4024-2

**Published:** 2017-02-14

**Authors:** Hendriek C. Boshuizen, Wilma J. Nusselder, Marjanne H.D. Plasmans, Henk H. Hilderink, Bianca E.P. Snijders, René Poos, Coen H. van Gool

**Affiliations:** 1 National Institute for Public Health and the Environment, P.O. Box 13720 BA Bilthoven, The Netherlands; 2 Wageningen University, Wageningen, The Netherlands; 3 Department of Public Health, Erasmus MC, University Medical Center, Rotterdam, The Netherlands

**Keywords:** Comorbidity, Disability weights, Incidence, Multi-morbidity, Prevalence, Risk factor attribution

## Abstract

**Background:**

Disability Adjusted Life Years (DALYs) quantify the loss of healthy years of life due to dying prematurely and due to living with diseases and injuries. Current methods of attributing DALYs to underlying risk factors fall short on two main points. First, risk factor attribution methods often unjustly apply incidence-based population attributable fractions (PAFs) to prevalence-based data. Second, it mixes two conceptually distinct approaches targeting different goals, namely an attribution method aiming to attribute uniquely to a single cause, and an elimination method aiming to describe a counterfactual situation without exposure. In this paper we describe dynamic modeling as an alternative, completely counterfactual approach and compare this to the approach used in the Global Burden of Disease 2010 study (GBD2010).

**Methods:**

Using data on smoking in the Netherlands in 2011, we demonstrate how an alternative method of risk factor attribution using a pure counterfactual approach results in different estimates for DALYs. This alternative method is carried out using the dynamic multistate disease table model DYNAMO-HIA. We investigate the differences between our alternative method and the method used by the GBD2010 by doing additional analyses using data from a synthetic population in steady state.

**Results:**

We observed important differences between the outcomes of the two methods: in an artificial situation where dynamics play a limited role, DALYs are a third lower as compared to those calculated with the GBD2010 method (398,000 versus 607,000 DALYs). The most important factor is newly occurring morbidity in life years gained that is ignored in the GBD2010 approach. Age-dependent relative risks and exposures lead to additional differences between methods as they distort the results of prevalence-based DALY calculations, but the direction and magnitude of the distortions depend on the particular situation.

**Conclusions:**

We argue that the GBD2010 approach is a hybrid of an attributional and counterfactual approach, making the end result hard to understand, while dynamic modelling uses a purely counterfactual approach and thus yields better interpretable results.

## Introduction

In 1993, the World bank report ‘Investing in Health’ [1] introduced the DALY as a measure for the burden of disease of a population. The DALY aims to “quantify loss of healthy years of life due to dying prematurely or to living with the health consequences of diseases, injuries or risk factors” [2]. This measure is operationalized as the sum of Years of Life Lost (YLL due to the mortality caused by a disease, injury or risk factor and Years Lost due to Disability (YLD) because of the disease, injury or risk factor.

DALYs were introduced to calculate the burden of diseases and injuries in a population. The YLL, YLD and DALYs of all individual diseases add to a total YLLs, YLDs and DALYs in a population. One important methodological critique on the DALY was that it took multi-morbidity insufficiently into account. When YLDs due to two different diseases are added, the years with multi-morbidity will contribute twice in the total burden in terms of YLDs. This is only valid if the disability weights of the two diseases can be added up, which is often not the case. Therefore, the 2010 global burden of disease study (GBD2010 [3, 4]) has improved the estimation of YLDs by attributing a smaller part to each disease in order to take this into account. This method eliminates any double counting of years with multi-morbidity in the total burden of disease in terms of YLDs, assuming the underlying assumption of independent disease occurrence is correct.

DALYs, however, are not only calculated for assessing the burden of diseases and injuries, but also for assessing the burden of specific risk factors [5]. DALYs of diseases and injuries are attributed to risk factors [5] using a counterfactual approach. In such an approach the burden of the risk factor is determined by comparing the current situation with a counterfactual situation where the risk factor is reduced to the minimum level. The counterfactual situation is calculated by applying population attributable fractions (PAFs) to the YLDs and YLLs of the diseases caused by the risk factor exposure, where the PAF is calculated from epidemiological studies. In many cases, one risk factor influences multiple diseases. In this case the DALYs prevented through different diseases are added up.

This attribution method for risk factors, as used for this in the GBD2010 (and as far as we can see in the GBD2013) is a mixture of an (using DALYs) and a counterfactual (using PAFs) approach. This results in an approach that can be criticized on three points, on which we will elaborate further in this paper: First, the PAFs that are used are calculated based on relative risks that apply to disease incidence, but in the PAFs are applied as if they apply to disease prevalence. Second, newly occurring morbidity in life years gained is ignored, which should not happen in a counterfactual approach. Third, the adjustments for multi-morbidity that were developed in the context of estimating the burden of disease do not work if the disease is not completely eliminated, which is the situation in burden of risk factor calculation where a risk factor, rather than a disease is eliminated and multiple diseases are influenced by the same risk factor.

In the Netherlands, burden of disease calculations are part of the Public Health Status and Foresight (PHSF) reports that are produced every fourth year. DALYs have been calculated since 1997 based on the prevalence method, and DALYs for risk factors were calculated with a method similar to that used in the GBD2010 in the 2002 PHSF Report. However, in preparing the 2006 PHSF Report, it was realized that the prevalence based method is problematic for estimating the burden of risk factors when using age-dependent relative risks and exposures [6]. Therefore in this and later documents [7, 8] a modelling approach was adopted, in which a multistate disease model is used to simulate what happens in a population when risk factor exposure is eliminated. To determine the burden of each risk factor in such a modelling approach, a multistate disease model is used to simulate a population, both “as is” (current situation) and after elimination of the risk factor (“what if”). In the simulated population one can observe what happens to the future years lived, both with and without elimination of the risk factor. The burden of the risk factor is then given by the difference.

However, when using a multistate modelling approach, the scale of the DALYs gained differs from that of the DALYs calculated with more conventional calculations. The reason for this is that the DALY calculates the gain from eliminating exposure in a single calendar year, while such a modelling exercise calculates the effect of eliminating exposure now and in all future years.

In order to obtain a DALY-measure on the same scale, one should model the effect of exposure in a single calendar year. This can be done by eliminating exposure only in the current calendar year, and afterwards returning it to what it would have been in the situation without the elimination.

We implemented this approach in the DYNAMO-HIA model, which is a generic software implementation of a dynamic multistate disease model, where users enter their own data. In this paper we compare this approach, using smoking as an illustration, with results using the conventional method used by the GBD2010. The aim of this work is to determine the magnitude of the differences in the burden of a risk factor that can result from applying different calculation methods and to obtain insight in the causes of such differences.

### Theoretical considerations on calculation methods for the DALY

In this section we will outline the calculation of the DALY, the conceptual problems with the method for attribution of the DALY to risk factors and describe the alternative method using dynamic modelling.

### Method of DALY calculation for diseases and injuries

**Fig. 1:**
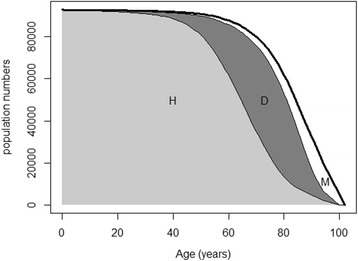
Concept behind the DALY attribution. On the y-axis the number of persons in the population that are still alive (healthy) at a particular age when following a birth cohort over time

The DALY is a measure for the burden of disease, which aims to “quantify loss of healthy years of life due to dying prematurely or to living with the health consequences of diseases, injuries or risk factors” [2]. Figure 1 shows the concept behind the DALY: the figure depicts the numbers lived in a population with and without disease related disability (area *D* and *H* respectively). In order to calculate the DALY, one first defines an envelope (here the thick black line) indicating the ideal situation: a situation without any burden of disease. In this ideal situation, the envelope would be completely filled with healthy years. In reality, however, there is a burden of disease, represented by the area M (the sum of years of life lost (YLL)) and the area D (the sum of years of life lost due to disability (YLD)). Now the aim of calculating a DALY for a particular disease or injurie is to attribute the areas *M* and *D* to the different diseases that cause the loss of health. As every person only dies once, the years contributed to *M* can be uniquely assigned to the primary cause of death of those dying prematurely. The GBD2010 developed a method (described in Appendix A) to attribute the years in *D* uniquely to each disease.

The DALY thus is operationalized as the sum of years of life lost (YLL) and years of life lost due to disability (YLD), where the latter is calculated as:

$$ \mathrm{Y}\mathrm{L}\mathrm{D} = D W* Y D, $$

where *YD* are the years that will be spend living with disease, and *DW* the disability weight.

There are two ways to calculate YD. The first, named the incidence-based method, estimates the YD by multiplying the incidence and the average period that an incident case is expected to live with the disease (to which we will further refer to as “duration of disease”). This method was initially used [1, 9]. The second, named the prevalence-based method, also referred to as the hybrid method [10], assumes a stationary situation. In that case the odds of the prevalence of the disease is equal to incidence times duration. Thus, when the prevalence is not too high, the YD can be estimated directly by using the prevalence of the disease in the population. This method is used in the GBD2010 and the current GBD2013. This latter method has the advantage of not requiring data on disease duration, which is hard to come by. Also the problem of multi-morbidity is more easily tackled.

### Method of DALY calculation for risk factors

In order to attribute DALYs to risk factors, a counterfactual approach based on PAFs is generally used. In a counterfactual approach one calculates the reduction in the burden of disease when exposure to a risk factor would have been at a theoretical minimum level. The PAFs are based on relative risks from the epidemiological literature on the associations of the risk factor with disease incidence or mortality. In the prevalence-based method, exposure refers to the past exposure, as the current prevalence of the disease is the result of past exposure of the risk factor. In the incidence-based method, exposure refers to the exposure that determines the incidence.

### Conceptual problems with the DALY calculation method for risk factors

In a counterfactual approach, the question is: what would happen if a disease or risk factor exposure would be eliminated? Such an counterfactual approach is conceptually different from a attribution approach, where the goals is to attribute each pixel in *D* and *M* in Fig. 1 uniquely to a single risk factor. Both approaches differ in the way they handle multi-causality. If, for instance, a disease only occurs when two causes are simultaneously present, the disease can be prevented by removing one cause, but just as well by removing the other cause. An attribution method will attribute only part of this disease to each cause (adding up to attributing the disease exactly one time in the total of all attributions), while a counterfactual approach will fully attribute the disease to each risk factor, because removing each risk factor will fully remove the disease. In other words, in this situation a counterfactual approach will attribute the disease twice. This is the reason for the well-known fact that PAFs can add up to more than 100%, or, more generally, that PAFs (and thus DALYs due to risk factors) are not additive.

In applying the PAF (representing a counterfactual approach) to disease specific DALYs (representing an attribution approach), the GBD2010 method for calculating the burden of disease due to risk factors is a mixture of two conceptually different approaches. The result is a hybrid method, the result of which is difficult to interpret.

There are several reasons why the method does not correctly represent the effects of elimination a risk factor (that is, setting a risk factor at the theoretical minimum level). The first and most important one is that with regards to the YLL, the method assumes that all years gained by eliminating mortality due to the risk factor will be healthy years. It thus ignores substituting morbidity [11]. In an attribution approach, where the goal is to attribute current morbidity and mortality, this is justified, but not in a counterfactual approach. This is because in a counterfactual approach, the years gained by elimination will be partly years spent in less than full health. Second, with regards to the YLD, the GBD2010 calculation implicitly assumes that multi-morbidity will remain the same after elimination of the risk factor. However, if part of the multi-morbidity is also prevented (because diseases share the risk factor), this is not the case and the method overcorrects. Third, the years lived with disease (YD, that is, the duration for a single incident case) are assumed to be constant. However, elimination of the risk factor will decrease mortality from diseases that share the risk factor, and thus YD will increase after elimination of the risk factor. This means that -in theory- risk factor elimination by decreasing mortality could even increase the YLDs. Lastly, as we show in Appendix A, the methods developed to take multi-morbidity into account, do not work in case of partial elimination of diseases (which is the case by elimination of one risk factor of a disease).

Another criticism on the method to calculated DALYs for risk factors is that the PAF is applied to YLDs calculated using the prevalence method. The PAF itself, however, is calculated using relative risks derived from studies on the association between risk factor exposure and disease incidence (or disease mortality), not prevalence. Therefore, this PAF should only be applied to YLD from an incidence and not a prevalence method. Applying the PAF to YLDs based on prevalence means that the ratio of disease prevalence in exposed and unexposed is assumed to be the same as the ratio of disease incidence in exposed and unexposed. In case of low prevalence and equal mortality in both groups, this might be approximately true, but only if exposure and relative risks are constant over age. Many exposures differ in older and younger age groups, and also relative risks generally decline with age. In that case the PAF that should be applied to prevalence-based YLD at a certain age group should be based on the exposure and relative risks at lower ages, at the moment that the disease first occurred (moment of incidence).

In summary, given a mix of a counterfactual and an attribution approach, and applying incidence based relative risks to prevalence, it is not clear what exactly the DALYs attributed to a risk factor represent.

### The alternative: dynamic modelling

In dynamic modelling, the evolution of disease in a population is simulated over time. This kind of modelling has been developed to perform counterfactual (“what-if”) calculations: The amount of disease and mortality in a counterfactual situation (here called a scenario) can be determined simply by rerunning the simulation under this scenario.

**Fig. 2:**
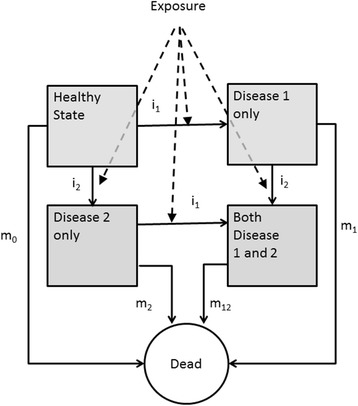
A multistate disease model for two diseases sharing an exposure. The transitions between states are governed by the incidence rates i (here taken independent of the presence of the other disease) and mortality rates m. In this model the exposure changes the incidence rates

Figure 2 presents such a model with two diseases. In this figure, the diseases are assumed to be irreversible, so no remission is included. Exposure to the risk factor influences the transition rate from a state without a particular disease to the state with a disease (the incidence rates i_1_ and i_2_, here assumed to be conditionally independent, that is, independent within those being equal with respect to risk factor status and presence or absence of disease). Furthermore, in every state subjects can die, governed by mortality rates m_x_ where the mortality rate depends on the state x (e.g. mortality is higher in those with the disease than in those without the disease). In this model, the probability of dying does not directly depend on the exposure, but does so indirectly, as exposure increases the incidence, and mortality is higher in those with a disease. The mortality in those without the disease is calculated from the population all-cause mortality by subtracting the disease-related mortality, which is calculated from the prevalence of the disease at baseline and the disease related mortality. The incidence rate of an individual with a certain risk factor exposure is calculated as a baseline incidence times the relative risk (RR) for that risk factor exposure. The relative risks are obtained from the literature, the incidence rates are based on data from GP or cancer registries.

Given a particular exposure, the accompanying incidence rates, and the effect of exposure on the incidence rates, the model can be used to simulate the future health states in a population.

DYNAMO-HIA is software (downloadable from www. Dynamo-hia.eu) simulating such a model for a single risk factor with a particular number of diseases [12, 13]. DYNAMO-HIA simulates the development of risk factor exposure over time using micro-simulation. Given the simulated exposure, it calculates the incidence rates and from that the probability of each health state over time. Part of the incident cases can die immediately when getting the disease (acute mortality).

In this model, a state is characterized by the presence or absence of each of the diseases that are modelled. Thus the number of states is 2^*N*^ were *N* is the number of diseases in the model, so multi-morbidity is explicitly modelled. After simulating the entire future development of disease states and death, the number of years lived within each health state in the population can be obtained by adding the probabilities of being in the state over all simulated individuals. These years then are multiplied by the appropriate disability weight. For each combination of diseases, a disability weight is calculated using a multiplicative formula, conforming to the same choice for a multiplicative effect as was made by the GBD2010 project:

$$ {\mathrm{DW}}_s=1-{\displaystyle \prod_d}\left(1-{\mathrm{DW}}_d\right) $$

where d is the index for the disease, DW_s_ the disability weight for the health state and DW_d_ the disability weight for someone with disease *d* only.

A change in the initial risk factor exposure, as for instance the complete elimination of exposure, changes disease incidence and in turn future disease prevalence and mortality. The gain in life years and in disability weighted life years from such elimination can be calculated by subtracting the (disability weighted) life years in a simulated population where the risk factor has been eliminated from those in a simulation where the exposure is equal to the observed exposure.

Such a calculation gives the gain for every person in the population over his or her entire future lifespan. In such a simulation one has to specify the exposure not only at the start of simulation, but also in future life years. If exposure is eliminated not only for the present, but also in the entire future, the gain will be much larger than that calculated by the DALY, that only aims to calculate the gain of eliminating exposure in a particular calendar year. In order to obtain a measure on the same scale as the DALY, therefore exposure is eliminated only in the current calendar year, and afterwards returns to what it would have been in the situation without the elimination. This is somewhat similar to the 10-year intervention suggested in 2003 in WHO-CHOICE’s generalized CEA methodology (WHO 2003) [14].

### Data and methods used for comparison of calculation methods

Our objective is to compare the dynamic modelling approach with the method used by the GBD2010 in order to obtain further insight in the magnitude of the differences between the two methods.

For illustration purposes we use smoking, in relation to four diseases: lung cancer, Chronic Obstructive Pulmonary Disease (COPD), Coronary Heart Disease (CHD) and stroke.

In order to make a fair comparison, we used the same data (incidence, mortality, relative risks etc.) in both methods. This means that the GBD method in this paper does not yield DALY comparable to those calculated by the GBD project, as they use different relative risk, mortality and prevalence data. For the same reason, we use the remaining life-expectancy for the Netherlands as calculated from the mortality rates used as basis for the YLL.

### Data sources used in the DYNAMO-HIA modelling

DYNAMO-HIA requires input on 1) demography (population numbers, all-cause mortality); 2) epidemiological information on disease incidence, prevalence, excess mortality (defined as the difference in mortality rate between those with and without the disease) and acute mortality for relevant diseases; 3) initial risk factor exposure and transition probabilities for risk factor exposure (in order to simulate future risk factor trajectories for the case with current exposure), and 4) relative risks linking exposure to disease. All these data are differentiated by age and sex.

In the illustration for this paper we used data estimates for the Dutch population with 2011 as the calendar year to which the calculations apply. Lung cancer incidence (1989–2011) and survival data (1989– 2010) were available from the Netherlands Cancer registry, and Poisson models (including time-trends) were used to model age- and gender specific incidence and excess mortality rates in 2011.

Stroke and coronary heart disease (CHD) incidence rates were taken from the Netherlands Information Network of General Practice (LINH) for 2011. For stroke and CHD, patients were linked to hospital records (1995–2010) in order to obtain only incident cases in those without previous CHD and stroke respectively, and linked to cause-of-death records from the national mortality register in order to include immediately fatal cases. Both prevalent and incident cases were linked to death records in order to obtain mortality for those with disease compared to those without the disease. For stroke and CHD, mortality was split into short term (less than 1 year) and long term mortality. Acute mortality was calculated by subtracting long-term mortality from the observed one-year mortality. COPD incidence data were from the same GP registration. Again both prevalent and incident cases were linked to death records in order to determine excess mortality rates.

Calculating death rates from differences between those with and without the disease has the advantage of including excess mortality where the disease is not registered as the cause of death. However, it might overestimate mortality in case there is confounding from risk factors (other than smoking, as that effect is adjusted for in our modelling). However, this potential overestimation will not disturb our comparison of methods, as this is used in both cases.

Although we also had current prevalence data for all diseases, for the purpose of this exercise we want to use a population in equilibrium, as we did not want our results to be influenced by time trends. In order to derive input data representing a population at equilibrium, we used the incidences and mortality rates described above and simulated the prevalence of disease in a cohort of newborns, who were all disease free at birth. The simulated future prevalence rates in this cohort were used as starting prevalence rates.

We used the smoking prevalence as obtained from a 2011 smoking survey from the Dutch Foundation on Smoking and Health in the population aged 16 and older. Smoking data comprised the proportion of current smokers, former smokers and never smokers. Smoking rates were assumed zero until and including age 10 and afterwards we let the rates increase linearly with age towards the observed prevalence rates at age 16. We used a multinomial spline model (package VGAM in R) to smooth the smoking rates over age. Again, as we want to use an equilibrium situation, we used transition rates that keep the age- and sex specific smoking probabilities constant over time.

We used the relative risks that were used for previous chronic disease modelling in the Netherlands, which smoothly change over age and are presented in Appendix A; we considered using the GBD2010 Relative risks [15] instead, but those are only available for smokers and non-smokers, while we prefer to distinguish former smokers from never smokers. Our relative risks differ by age. For cardiovascular disease they decrease with age, as is also the case in those used for the GBD2010 [5]. This is due to the fact that the same absolute risk difference translates to a lower relative risk when the baseline risk (that is, the risk in never smokers) increases with age. For COPD and lung cancer, the relative risks first increase with age, reflecting higher average cumulative exposure with age, but at high ages the effect of increasing baseline risk takes over and relative risks decline with age [16].

In order to obtain insight in the causes of the differences between methods, we additionally performed analyses using an artificial situation (artificial data instead of the empirical data described above) in which:

- relative risks do not depend on age (see Appendix A for the values used)
- smoking proportions are the same for all ages, and subjects do not change their smoking status over age
- disease prevalence rates are in equilibrium with the mortality and incidence rates under these new conditions
- a stable population: the age distribution is in equilibrium with the mortality rates (under a constant birth rate).

In this last artificial situation, the prevalence method and incidence method are expected to deliver more or less similar results, so the difference between the dynamic modeling and the GBD2010 method will not be due to these differences.

We used the following disability weights: cancer of the lung: 0.285; stroke 0.609, CHD 0.288 and COPD 0.314. The values were derived assuming that the distribution over severity states of each disease is fixed and are equal to those used in the 2014 PHSF report [8].

In the DYNAMO-HIA model, also a disability weight is given to those without one of the diseases in the model, as these persons will have disabilities due to diseases that are not modelled, or simply due to old age. We both ran the analyses with and without these extra disability weights. In the latter case perfect health is assumed for anyone without one of the four diseases in the model.

### Data manipulation for the use of data in the GBD2010-type calculations

In order to have a fair comparison, the data used in the GBD2010 method should be the same as those used in the DYNAMO modelling. Below we describe how we obtained such data.

### Mortality (YLL)

Disease specific mortality in the DYNAMO model is not obtained from statistics on cause-specific mortality, but from the difference in mortality between those with and without a disease (which DYNAMO-HIA names “excess mortality”), adjusted for effects of risk-factor related multi-morbidity from other modelled diseases [17]. In order to use mortality rates in the GBD2010-type calculation equal to the data used in the DYNAMO-HIA modelling, we used this attributable mortality.

The mortality rate used was calculated as follows: First, the attributable mortality rate in the prevalent population was taken from the model. From this the one-year probability of dying was calculated as *1-exp(- m*_*a*_ ) + i * (1-(1-exp(- m_*a*_ *))/m*_*a*_ *),* where *i* is the incidence rate (excluding fatal cases), and *m*_*a*_ the attributable mortality rate. The last term in this expression represents the mortality from those who acquire the disease during the simulated year, and die before the beginning of the next year. For diseases with acute mortality (stroke, CHD) we added the mortality of newly diagnosed cases to this mortality rate.

As the number of life years lost for each death we took the remaining life expectancy for that age and gender. By doing so we deviate from the GBD2010 method, which used a normative life table. For this exercise, however, our choice makes for a better comparison between methods, as the same life table is used in both methods.

### Prevalence (YLD)

The disease prevalence was the same as used as initial prevalence in the DYNAMO-HIA model.

We multiplied the disease prevalence in our population with the PAF calculated based on the relative risks and risk factor exposure prevalence and the disability weights as used in the DYNAMO-HIA model. The disability weight was adjusted for multi-morbidity using the same method as applied in the GBD2010, and described above. However, as we only used four diseases, we could do so by straightforward enumeration instead of simulation.

## Results

Table 1 shows the YLLs calculated according to the GBD method and based on the overall years gained by elimination the risk factor from the DYNAMO-HIA model. The DYNAMO-HIA model is not able to attribute death to specific causes as death, so only total years gained are available. In the illustration case, the YLLs calculated by dynamic modeling are smaller than those by the GBD2010 approach. In contrast, in the artificial case (with exposure and RRs equal for all ages), the YLLs calculated by dynamic modeling are larger than those calculated by the GBD2010 approach.

**Table 1:** Years of Life Lost (YLL; in thousands) calculated with the GBD2010 method and with DYNAMO-HIA. Both with realistic data (Illustration case) and with artificial data

Disease		lllustration Case	Artificial data^a^
Dynamic Modeling			
	Men	187	173
	Women	138	127
GBD method			
Lung cancer	Men	68.7	66.7
	Women	63.4	55.9
CHD	Men	23.3	47.4
	Women	9.1	23.3
Stroke	Men	12.0	24.8
	Women	8.6	22.7
COPD	Men	58.8	63.1
	Women	39.0	44.1
Total	Men	163	202
	Women	120	146

^a^the risk factor exposure and relative risks are the same for all ages, and the population is stable over time

Overall, however, the differences between the results from the different methods were less than 20%.

Table 2 shows the YLDs calculated with the GBD2010 method compared to the values obtained when subtracting the YLLs from the dynamic modelling from the DALY values obtained from Dynamic modeling. The DYNAMO-HIA model does not split the disability in those with multiple diseases in parts attributed to individual diseases. Therefore DYNAMO-HIA is not able to attribute YLDs to specific diseases, so only total healthy years gained are available. Here lower “YLD” values in the dynamic modelling compared to the GBD2010 approach are observed for all cases where disability from all diseases is included in the dynamic modelling.

**Table 2:** Years Lost to Disability (YLD; in thousands) calculated with the GBD2010 method and with DYNAMO-HIA. The latter is calculated as DALY minus YLL. Both with realistic data (Illustration case) and with artificial data

		Illustration Case	Artificial data^a^
Daly – Yll from Dynamic Modeling			
Including DW from other diseases	Men	68	51
	Women	54	47
Only DW from smoking- related diseases	Men	99	82
	Women	88	80
YLD GBD2010 method			
Lung cancer	Men	3.5	3.8
	Women	2.6	2.5
CHD	Men	24.6	48.5
	Women	16.9	37.2
Stroke	Men	13.7	23.7
	Women	9.8	22.6
COPD	Men	60.7	61.0
	Women	54.7	59.2
Total	Men	123	137
	Women	84	122

^a^the risk factor exposure and relative risks are the same for all ages, and the population is stable over time

With a zero disability weight for the non-smoking related diseases to model the situation of less substitute morbidity (last lines under dynamic modeling in Table 2), the dynamic modeling “YLD” will comprises only “healthy years gained” related to the four diseases included in the model. Here the difference with the GBD2010 method is considerably smaller, and for women the healthy years gained from the dynamic approach are even slightly larger than the YLDs calculated with the GBD2010 approach.

In the artificial case (with exposure and RR not depending on age) the years gained from decreased disability in the dynamic approach are considerably lower that the YLDs calculated by the GBD2010 method.

Together this means that the GBD method assigned 470,000 DALYs to smoking, while the dynamic modelling method yielded 447,000 DALYs, which rose to 512,000 DALYs when disability of non-smoking related diseases was ignored. Although these final figures are rather similar, the artificial calculations show that these figures hide larger but opposing differences that partially cancel each other out. In the artificial data the GBD method assigned 607,000 DALYs to smoking, and the dynamic modelling 398,000, rising to 462,000 when disability of diseases not related to smoking was ignored.

## Discussion

Our case study shows that the DALY calculated with conventional methods overestimate the gain that can be obtained by eliminating exposure to a risk factor. There are several explanations behind this.

First, the GBD2010 methods assumes that all years saved by eliminating mortality will be completely healthy years, while in the dynamic modeling approach they will be partly years lived with disability.

This is seen most clearly when in the dynamic modelling DALY weights are used not only for the four diseases explicitly modeled, but also for all other (not risk factor related) diseases (Table 2).

A second explanation is that age-varying risk factor exposure or relative risks also lead to fairly large discrepancies between results using the GBD2010 method and dynamic modelling with DYNAMO-HIA. This is shown most clearly by comparing the calculations with age-constant exposure and relative risks (artificial data, Tables 1 and 2). The GBD2010 method uses relative risks, smoking prevalence and the population numbers at a particular age *a* to calculate the eliminated mortality at age *a*. In contrast, in the dynamic modeling the mortality at age *a* is influenced by relative risks and exposure at younger ages. As we used relative risks as well as smoking rates that decline mostly at higher ages (Appendix A), this results in a higher YLL for the dynamic modelling approach.

We expect these differences will also show when an incidence-based method would be employed for DALY calculations (also eliminating exposure effects on new cases only) instead of a prevalence based method, eliminating future effects of current exposure rather than current effects of past exposure.

However, a more fundamental problem is that the GBD2010 method is a mixture of two conceptually different approaches. The DALYs for diseases in the GBD2010 clearly implements an attribution approach, aimed at unique attribution of DALYs to diseases. In the attribution of risk factors, however, this is mixed with a counterfactual approach. The result is a hybrid method, the result of which is difficult to interpret.

We propose a consistent counterfactual approach for estimating the burden of disease of risk factors. In this proposed approach, a multistate disease model is used to calculate the health gains of eliminating exposure in a single year. The advantage is that it is conceptually clear what is calculated. In order to deliver numbers pertaining to a single calendar year, we propose to eliminate the exposure only in that particular calendar year, and afterwards return the exposure to what it would have been without the elimination.

The dynamic modelling approach with DYNAMO-HIA depends on assumptions: Incidence and mortality rates are assumed to be stationary; duration of survival in those with the disease is assumed not to depend on risk factor exposure; eliminating exposure is assumed to immediately reduce the risk; and it is assumed that the dose response function between exposure and disease incidence can be described perfectly by a relative risk. This will make the modelling only a crude approximation of reality. The modelling therefore is not suitable for delivering predictions of future health, but is meant as a method for “what-if” calculation, in this case: what if exposure would be eliminated? As similar assumptions are made in the GBD project, we do not believe these assumptions make our method less reliable than those calculations.

In DYNAMO-HIA we do not model phases and sequelae of each disease separately, but apply the same disability weight to everyone with the disease. In principle, however, dynamic modelling could be extended to including disease stages.

In the GBD2010, the incidence method of calculating DALYs from earlier GBDs was replaced by a prevalence-based -or better: hybrid [10] – method. This made it possible to refine the YLD calculations with co-morbidity adjustments that make this method into a consistent attribution methodology. One of the virtues of the prevalence-based DALY is its simplicity: it only takes estimates of prevalence and mortality to enable a reasonable estimate of the burden of disease. Dynamic modelling is more complex and requires more data and knowledge. For looking at risk factors, however, the simplicity of the GBD method comes with the price that it is not completely clear what entity is calculated.

In GBDs before the GBD2010, the incidence-based method was used, and a choice between attribution or counterfactual approach was less clearly made. The use of population attributable (or preventable) fractions is much more natural with the incidence-based method, as the relative risks on which the preventable fractions are based are almost exclusively taken from studies on incidence (or mortality) of disease. The reason that incidence is generally the preferred outcome to study as an effect of risk factor exposure is because causality can be more easily assumed in case of incidence. For instance, an observed relation between obesity and prevalent osteoarthritis can also be explained by “reverse causality”, that is, pain from osteoarthritis promotes a sedentary life style which leads to obesity.

Applying a relative risk for incidence to prevalence needs justification. One justification could be that with low prevalence rates and when disease-related mortality rates are independent of the risk factor, the prevalence ratio will be approximately equal to the incidence rate ratio. However, this assumes that exposure is constant over time, and relative risks are not age dependent. Our calculations showed that when this is not the case, effects on the result are not negligible. Lette et al. [18] give a method translating incidence-based relative risks into prevalence ratios, but this method has not been used in the context of prevalence-based DALYs.

It is often stated that “without discounting or age weighting, incident and prevalent YLD for a disease should be equal if there have been no past trends in incidence, mortality or remission” [2]. However, this is incomplete, as there also should be a stationary population in terms of size and age structure. Moreover, it does not apply when calculating the burden of disease from risk factors: in that case the requirement is also that there should be no time trends or age-related trends in exposure and relative risks. In most cases this is unrealistic.

When using age-constant relative risks and exposure, we see lower YLLs in the dynamic modeling (Table 1). This difference is caused by the built-in delay time between incidence of disease and mortality from that disease. This delay originates from the fact that exposure influences only disease incidence directly. Mortality is only increased after the prevalence of the disease has increased. Here one might argue that this effect is an artefact of the assumptions build into the model. However, such a delay is not unrealistic, and therefore constitutes a less strong assumption than assuming no delay.

Although the YLL from the dynamic modelling approach is higher than that obtained with the GBD2010 approach, the DALYs are lower. An important reason is that the life years gained in our model are not assumed to be all healthy years, but are part of the simulated population, and thus partly spend with (new) disease. This means that in a counterfactual approach the YLLs are only partly healthy years. From a more conceptual point of view, one could argue that this is because in the counterfactual approach one considers the gain in the neighbourhood of the current situation, while the attribution approach contrasts the current situation with an ideal situation where all diseases have been eliminated.

In the GBD2010 method, the magnitude of the YLLs is for an important part determined by the choice of the envelope used to determine how long someone saved from death still has to live. This choice is arbitrary. A different choice will influence the DALYs of risk factors associated with lethal diseases proportionally more than those that mostly cause disability. For comparison reasons we used a country-specific envelope, narrower than that used in the GBD. Using the GBD envelope would have made the overestimation due to the assumption that all life years gained are healthy years even larger. An advantage of a true counterfactual approach is that it is no longer necessary to define a mortality envelope, as the gain in life years is automatically calculated based on the current mortality rates and the mortality rates after elimination of the risk factor.

Although different methods give different results, one could ask whether this is of practical relevance in comparative studies, especially as the expertise and data resources needed to prepare input data for dynamic models might not be universally available. Although methods might deliver different absolute numbers, they could still yield similar rankings between countries or risk factors. Things as increase of relative risks, incidence and mortality with age are universal, and therefore their influence on the ranking of results between countries might be limited. On the other hand, when there are clear survival differences between countries (leading to a different composition of the prevalent cases in terms of disease duration), or differences in the distribution of exposure over age groups, or where populations have a different age-structure, the ranking might be affected. Also risk factors differ with regards to which age groups are mostly exposed, so there is clear potential for interference with rankings, especially where estimates are not too far apart.

In summary, we argued that the dynamic modelling approach follows a clear conceptual approach and thus is easier to understand in terms of what is being calculated. Also aspects as multi-morbidity and substitution of morbidity and mortality are automatically incorporated. As one strives to more accurate methodology, we believe use of such models is indicated rather than devising ever more sophisticated adjustment methods for DALY calculations.

## Figures and Tables

**Fig. 3:**
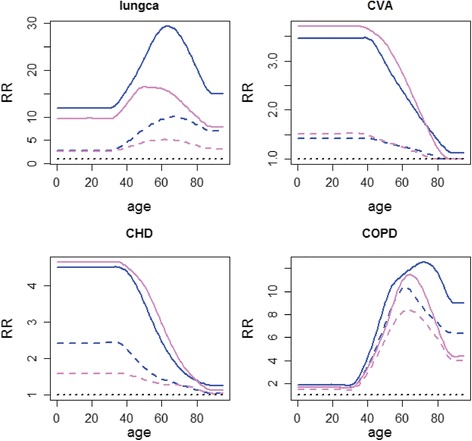
age dependent relative risks used in the simulation for smokers and former smokers in men and women. *Blue* = males, *pink* = females; full line: current smokers, dashed line: former smokers

**Fig. 4:**
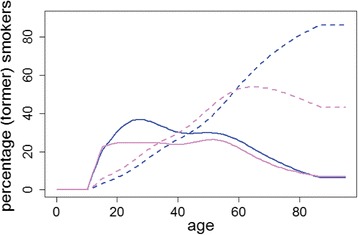
Percentage smokers and former smokers in Dutch men and women used in the realistic simulation. *Blue* = males, *pink* = females; full line: percentage current smokers, dashed line: percentage former smokers

## Appendix A

### Consequence of applying the GBD2010 method for multi-morbidity adjustment in risk factor elimination

In this appendix we will work out the GBD2010 method for multi-morbidity adjustment for two diseases A and B, and show that this adjustment delivers the correct amount of eliminated disability weighted years when both diseases are completely eliminated, but not in most other cases where a disease is only partially eliminated.

Let the unadjusted disability weights for the diseases A and B be equal to D*W*_*A*_ and D*W*_*B*_ respectively, and the weight adjusted according to the GBD2010 methodology be D*W*_*Aadj*_ and D*W*_*Badj*._ The unadjusted weights are valid for someone with only disease A (or only disease B), while the adjusted weights are those applied in the end to every person with disease A (or B).

In order to simplify the equations, we introduce the “ability weights” *A*_*X*_ = *1-DW*_*X*_ with *X* = A or B.

The GBD2010 (as well as the DYNAMO-HIA model) uses a multiplicative model for determining the disability weights in case of multi-morbidity, which implies that the ability weight for someone with both disease A and B is equal to the product of the abilities for disease A and disease B:

$$ {A}_{A B}={A}_A{A}_B $$

This assumption of multiplicativeness is only an assumption, and other assumptions are possible. However, this falls outside the scope of this paper.

In case of 2 diseases, the ability in the population is:

$$ {A}_A\left[ p(A)- p\left( A{\displaystyle \cap } B\right)\right]+{A}_B\left[ p(B)- p\left( A{\displaystyle \cap } B\right)\right]+{A}_A{A}_B p\left( A{\displaystyle \cap } B\right) $$

Where *p(A)* and *p(B)* represent the prevalence of disease A and B respectively and *p*(*A* ∩ *B*) the prevalence of having both disease A and B.

In the case that the prevalence of A and B are independent (thus *p*(*A* ∩ *B*) = *p*(*A*)*p*(*B*)), this is equal to:

$$ {A}_A p(A)+{A}_B p(B)-\left({A}_A+{A}_B-{A}_A{A}_B\right) p(A) p(B) $$

The adjustment carried out by the GBD splits up the last term up in two parts which are incorporated into *A*_*A*_ and *A*_*B*_ respectively, yielding an adjusted version of *A*_*A*_ and *A*_*B*_ in such a way that

1 $$ {A}_A p(A)+{A}_B p(B)-\left({A}_A+{A}_B-{A}_A{A}_B\right) p(A) p(B) = {A}_{A_{adj}} p(A)+{A}_{B_{adj}} p(B) $$

There are multiple ways to split-up the last term into two parts. The GBD has chosen to split up the weight 1-*A*_*A*_ *A*_*B*_ for someone with both diseases in proportion to D*W*_*A*_ and D*W*_*B*_. For the argument here, however, the way of splitting is not relevant.

Now assume that a fraction x of disease A is eliminated, and a fraction y of disease B. In our application (elimination of risk factors) these fractions are the preventable (or population attributable) fractions (PARs).

The ability in the population after elimination is equal to:

2 $$ \left(1- x\right){A}_A p(A)+\left(1- y\right){A}_B p(B)-\left(1- x\right)\left(1- y\right)\left({A}_A+{A}_B-{A}_A{A}_B\right) p(A) p(B) $$

In the methodology of the GBD2010 the adjusted version of the (dis)ability weight is applied to this case also, thus the ability after elimination of the risk factor according to the GBD2010 method is equal to:

$$ \left(1- x\right){A}_{A_{adj}} p(A)+\left(1- y\right){A}_{B_{adj}} p(B) $$

where A_A_adj_ and A_B_adj_ have the same adjusted value as are used for calculating the disease YLDs. In order for this method to work, this should be equal to [2].

Slightly rearranging this equality and equation [1], this means that in order for the GBD2010 method to work, the following two equations should be valid simultaneously:

$$ \left({A}_A-{A}_{A_{adj}}\right) p(A)+\left({A}_B-{A}_{B_{adj}}\right) p(B)=\left({A}_A+{A}_B-{A}_A{A}_B\right) p(A) p(B) $$

3 $$ \left(1- x\right)\left({A}_A-{A}_{A_{adj}}\right) p(A)+\left(1- y\right)\left({A}_B-{A}_{B_{adj}}\right) p(B)=\left(1- x\right)\left(1- y\right)\left({A}_A+{A}_B-{A}_A{A}_B\right) p(A) p(B) $$

and thus

$$ x\left({A}_A-{A}_{A_{adj}}\right) p(A)+ y\left({A}_B-{A}_{B_{adj}}\right) p(B)=\left( x+ y- xy\right)\left({A}_A+{A}_B-{A}_A{A}_B\right) p(A) p(B) $$

For each chosen value of x there is only a single value of y for which this equation will hold, for all other values of y the adjustment will not yield the right result. As y and x are given by the PAF for risk factor elimination, it is unlikely that they will satisfy this condition.

Note that the method will work for complete elimination of both diseases (x = y = 1), but not for complete elimination of only one disease (e.g. x = 1 and y = 0), or partial elimination of a disease.

Also note that the GBD method above is based on the assumption that the probability of each disease is independent. However, in calculating a PAF, the diseases will share a risk factor, and because of this they will be dependent.

Tables 3 to 5 in Appendix A illustrate the theory above with a simple numerical example. In this example we take a population of 10,000 persons (of the same age and gender) and let disease A and B both have a prevalence of 10% (=1000 persons), and a disability weight of 0.3.

**Table 3:** Simple example: Situation in the population without any elimination

	N in Population	Disability weight	Disability Weighted N	Adjusted Disability weight	Adjusted Disability weighted N
Healthy	8,100	0	0	0	0
Disease A only	900	0.3	270	0.2955^1)^	265.95
Disease B only	900	0.3	270	0.2955^1)^	265.95
Both disease A and B	100	0.51	51	0.591^2)^	59.1
Total	10,000		591		591

^1)^(0.51/2 *100 + 0.3*900)/100 = 0.2955

^2)^0.2955*2

**Table 4:** Simple example: population after elimination of disease A only

	N in Population	Disability weight	Disability Weighted N	Adjusted Disability Weight	Adjusted Disability weighted N
Healthy	9,000	0	0	0	0
Disease A only	0	0.3	0	0.2955	0
Disease B only	1,000	0.3	300	0.2955	295.5
Both disease A and B	0	0.51	0	0.591	0
Total	10,000		300		295.5

**Table 5:** Simple example: population after elimination of 50% of disease A and 50% of disease B

	N in Population	Disability weight	Disability Weighted N	Adjusted Disability Weight	Adjusted Disability weighted N
Healthy	9,025	0	0	0	0
Disease A only	475	0.3	142.5	0.2955	140.3625
Disease B only	475	0.3	142.5	0.2955	140.3625
Both disease A and B	25	0.51	12.75	0.591	14.775
Total	10,000		297.75		295.5

When both diseases are completely eliminated, we eliminate 1000 cases of disease A and 1000 cases of disease B, both having an adjusted DALY weight of 0.2955 (Table 1 in Appendix A). So we eliminate 2 times 295.5 is 591 YLDs. This is exactly equal to using the method that writes out the disability per group (Table 1 in Appendix A, column “Disability weighted N”). When one disease is eliminated (Table 2 in Appendix A), or the two diseases are partly eliminated (Table 3 in Appendix A), the adjusted method delivers a lower remaining disability in the population (as both diseases are present before elimination), and thus a higher estimate of the potential gain. The example also shows that under these conditions, both the effect of the adjustment, and the errors made in the adjustment are small.

## Appendix B

### Input data of the model

#### Relative risks used

Figure 3 in Appendix B gives the age-depend relative risks used in the calculations. When constant relative risks were used, we used for current smokers the RR from the GBD2013 for lung cancer (22.51 and 14.10 for men and women respectively) and for COPD (11.44 and 15.24 for men and women respectively), while for former smokers we arbitrarily took 7 and 4 for lung cancer and 10 for COPD (for both sexes). For CHD and stroke we used the same RRs in both sexes: 3.5 for current smokers and 1.5 for former smokers for CHD and 2.5 (current smokers) and 1.2 (former smokers) for stroke.

#### Smoking rates

Figure 4 Appendix B presents the age-dependent smoking rates used in our realistic simulation.

## Abbreviations

CHD: Coronary heart disease
COPD: Obstructive pulmonary disease
DALY: Disability adjusted life years (lost)
GBD2010: Global burden of disease 2010 study
PAF: Population attributable fraction
RR: Relative risk
YLD: Years Lost due to disability (caused by a disease, injury or risk factor)
YLL: Years of life lost (due to the mortality caused by a disease, injury or risk factor)
